# The opportunities and challenges of virtual coach education in rural and remote communities

**DOI:** 10.3389/fspor.2025.1644718

**Published:** 2025-12-12

**Authors:** Carrie W. LeCrom, Kalyn McDonough Smith

**Affiliations:** Center for Sport Leadership, Virginia Commonwealth University, Richmond, VA, United States

**Keywords:** sport for development, coaches, coach education, virtual education, task–technology fit

## Abstract

**Introduction:**

Within sport for development (SFD), it has been widely established that coaches—and the presence of strong adult relationships—play a critical role in facilitating a broad array of positive youth outcomes. However, the ability of coaches to play a meaningful role is influenced by their involvement in coach education and the support they receive from learning communities. While coaching education is important, not all coaches can access it readily, due to a lack of programs in certain geographic areas, a lack of local/in-person expertise, financial or time constraints, and other factors that make it inaccessible. Virtual education has emerged as a potential strategy to overcome these challenges, but despite growing technological adoption, practice has outpaced research, and evidence supporting the efficacy of virtual coaching remains limited.

**Methods:**

Guided by the Task–Technology Fit Framework, this study assessed virtual coach education across ten sport-for-change organizations in ten countries to illustrate and understand access, strategies, strengths, and limitations associated with virtual coach education.

**Results:**

Findings showed high motivation among the sport-for-change organizations to integrate virtual coach education into their coach education strategies and overall capacity building, revealed variation in the utility of virtual technology among organizations, challenges to technological use related to access and resources, and finally strategies employed to support learning in a virtual space.

**Discussion:**

Overall, the current study revealed a major equity issue in relation to SFD organizations engaging in virtual coaching training, highlighting the influence of structural inequities which continue to cause barriers to technology access that can exacerbate the equity gap. As is often the case, the communities that face the greatest marginalization experienced significant inequities in access to quality coach education. Building on creative strategies outlined in the article, it is recommended that researchers and practitioners continue to think “outside the box” on virtual coach education strategies as well as the broader role of coach education.

## 1 Introduction

Within sport for development (SFD), a continued concentration of research examines the role of sports in youth development (1) and in supporting youth in building, practicing, and transferring valuable life skills off the field (2–4). Across various contexts and youth populations, scholars have developed a well-established line of inquiry into the utility of sport, specifically with young people from marginalized communities. This includes using sport to support positive youth and life skill development (5, 6), social inclusion (7), healing among youth who have experienced complex trauma (8), and sport as a mechanism for social justice for those who have experienced inequitable privilege–power–oppression dynamics (9, 10), among others. Yet, across these different bodies of work, a constant thread is present—the critical role of coaches and strong adult relationships in facilitating such outcomes through sport.

Looking specifically at the literature on positive youth development (PYD) and life skills through sport, youth sport coaches have been found to play an essential role in the creation of constructive program environments, which help young people build healthy relationships with their coaches and one another, as well as facilitating a safe space for learning and practicing valuable life skills (1, 9, 10). Earlier work has identified the importance of intentionality in how coaches teach such skills and in building a “PYD climate” (1). For instance, Bean et al. (2) developed an implicit/explicit continuum of life skills development and transfer, outlining six levels through which coaches can facilitate such processes: (1) structuring the sport context, (2) facilitating a positive climate, (3) discussing life skills, (4) practicing life skills, (5) discussing transfer, and (6) practicing transfer. Yet, Camiré et al. (9) argue that the ability of coaches to play a meaningful role in these processes is often influenced by their involvement in coach education around PYD and the support they receive from learning communities. In more recent years, researchers have raised critical concerns regarding the limitations of the PYD framework in acknowledging systemic issues of power, privilege, and oppression in the lives of young people.

A fundamental critique of PYD and conventional coaching techniques is that they have not accounted for oppression, discrimination, and marginalization in the lived experience of youth athletes across communities (11). Thus, a critical positive youth development framework emerged that positioned critical consciousness within the existing PYD framework as a means to empower all young people in understanding their own personal experiences and those of others within unjust systems and support collective action toward social change (11). This call to center the voices of those who have been traditionally marginalized, along with more critical youth development approaches, has fueled a wave of work among youth sport researchers and practitioners (12, 13). Camiré et al. proposed that a critical perspective could help “reimagine” PYD and life skills in youth sport, whereby life skills (1) take on expanded meanings, (2) are viewed as sociopolitical, and (3) address the social realities of youths (9). Within this body of work, the valuable role that coaches play in bridging toward social justice life skills has been emphasized (14), as well as the necessity of supporting coaches in building their own social justice skills through coach education (10).

Assessing other bodies of work in SFD among youth populations, Morgan and Parker (7) analyzed the impact of sport-based interventions for youths who had been deemed at risk, were gang-affiliated, and/or had justice involvement. Participants reported feeling a greater sense of social inclusion and belonging after being involved in the program, but authors noted that these findings were largely contingent upon “contextual conditions,” the most important of which was young people having a strong, trusting relationship with key program personnel such as coaches. Similar findings were outlined in relation to the role of sport in supporting healing from trauma among young people, wherein this opportunity was contingent on the presence of supportive relationships (coaches, mentors, peers) (8). Thus, looking at the expansive work done in youth sport and on youth sport coaches, it has been well established that the (broad) experiences of young people within sport programs are deeply impacted by coaches (15). Specifically, it has been noted that the knowledge, attitudes, and behavior of coaches (15, 16), and their ability to build trusting, supportive relationships (7), ultimately play a significant role in facilitating beneficial outcomes for young people (16).

In order to support coaches in such an integral role, coach education and professional development have been found to increase coaching skills and effectiveness (16, 17), as well as their ability to empower positive youth sport climates, as mentioned previously (9). Nelson et al. (18) outlined three distinct categories of coach education program interventions (CEPIs): formal, informal, and non-formal. Whereas formal interventions can include certification programs through national sport associations, informal interventions typically describe personal experiences, and non-formal entails structured educational activities that are not endorsed through any one organization but intended to share relevant educational information (i.e., seminars, workshops, clinics). In a systematic review and meta-analysis of the effectiveness of coach education on coaching effectiveness in youth sport, Li et al. (16) noted that positive effects of CEPIs were observed in 78% of studies. These effects included enhanced knowledge, shifts in attitude and behavior changes among coaches, and improved physical and psychological outcomes for youth on and off the field.

While it has been established that coaching education is important, not all coaches can access it readily due to a lack of programs in certain geographic areas, a lack of local/in-person expertise, financial or time constraints (19–21), and other factors that make it inaccessible. As is often the case, the communities that face the greatest marginalization—and are the primary focus of SFD programs—also experience similar inequities in access to quality coach education (22). To overcome these barriers, virtual or hybrid education has been identified as a potential strategy for broadly disseminating foundational coaching knowledge and skills (16).

As a result of technological advancements, and environmental circumstances such as the COVID-19 pandemic, online and technology-enhanced methods have emerged as innovative mediums for coach education (23). Driska and Nalepa (24) reported that, over the past 20 years, coaching organizations have increasingly utilized online and self-paced delivery, but reliance on online delivery increased exponentially as a response to in-person restrictions during the global pandemic (24, 25). This “new normal” now appears to be firmly embedded in coach education strategies among national sport federations, youth sport organizations, and higher education institutions (23). The benefits of online coach education include widespread reach and fidelity, low cost, and reduced burden on coaches (24). Additional advantages include enhanced ease of access to information and greater uniformity of content across regions (26). Despite the enhanced use of technology in coaching, teaching, and learning, the practice has outpaced research in this field, and initial reviews of the literature have found only weak support for its efficacy (27). Cushion and Townsend (27) noted that there is a pressing need to better understand how technology is currently used in coach learning as well as the impact of its use.

In reflecting on the literature, our own experiences as two SFD researchers engaged in applied programming largely mirrored these current sentiments. We observed a rise in government- and foundation-sponsored grant applications that required a virtual component. Through our work in sport diplomacy and international coach education for sport-for-social-change organizations, we have utilized virtual trainings in a variety of ways: as follow-up sessions after in-person trainings, as monthly roundtable discussions for knowledge exchange, and as virtual spaces to build community among past and existing partners. The ability to connect with partners in Asia or Africa using Zoom or Teams and conduct a two-hour coaching training—which would otherwise require 20–30 h of travel—was undeniably convenient and economical. Yet, as with other researchers, we grappled with questions of effectiveness, quality, and impact, alongside concerns about equity, accessibility, resources, and technological availability when working with rural and remote communities. Operating in a post-pandemic world, these questions provided an opportunity to pause and reflect on whether, under what circumstances, this universal momentum toward virtual coach education is useful, and whom it ultimately serves?

Therefore, we conducted this research as an opportunity to understand how SFD organizations across the globe are engaging with virtual coaching education. Specifically, we sought to examine what practices are working and what are not, which platforms have demonstrated success, how programs are assessing the effectiveness of virtual coach training. The following research questions guided this study: (1) How have SFD organizations approached virtual education? (2) What are the opportunities and challenges of virtual coach education among SFD organizations? (3) What strategies have SFD organizations implemented in virtual coach education?

## 2 Methods

Given the exploratory nature of the study, a basic qualitative design was adopted, focusing data collection around interviews that took a post-positivist philosophical approach (28, 29). Post-positivism is an approach that pursues evidenced-based truths through inquiry, while acknowledging that there is no absolute truth (28, 29). Post-positivist research is widely used in educational settings in an attempt to explore a phenomenon and create meaning. Through a combination of convenience and snowball sampling, ten SFD program administrators or directors were recruited for interviews. As this is an exploratory piece, the strategic decision was made to interview program administrators instead of coaches to understand what is being done currently at the organizational level. The research team began by utilizing their own network of SFD colleagues across the globe, choosing only those organizations that self-reported engagement in some form of virtual coach training/education in rural or remote communities. Those interviewed also suggested additional SFD organizations engaging in this work, rounding out the sample. Being strategic in geographic diversity, the sample resulted in ten SFD organizations in ten different countries, in the Global South and the Global North, with most organizations operating in rural or remote communities. Although a standardized definition of “rural” and “remote” is not largely agreed upon in the literature, several characteristics have been identified to help operationalize these multidimensional concepts. These include geographic isolation, lack of social services, thin economies, and limited public transportation (30). Sports utilized in programming included athletics, basketball, frisbee, flag football, karate, lacrosse, roller skating, soccer, and tennis, with some organizations working across multiple sports. Table 1 outlines organization demographics, including geographic location, sports utilized, and full-time staff size.

**Table 1:** Participant organization demographics.

Organization	Geographic location	Sport	Full-time staff size
A	West Africa	Multi-sport	2
B	Sub-Saharan Africa	Soccer	4
C	Sub-Saharan Africa	Multi-sport	6
D	Southeast Asia	Multi-sport	6
E	North America	Soccer	11
F	South Central Asia	Soccer	14
G	North America	Lacrosse	14
H	Central Africa	Soccer	16
I	Europe	Multi-sport	25
J	Oceania	Multi-sport	130

The interview guide was created utilizing Task–Technology Fit (TTF) Theory, which emphasizes a critical construct in information systems and performance in that technology can have a positive impact only if it “fits” the task for which it is being used (31). As the focus of our study is on SFD organizations working in rural and remote communities, the significance placed on the fit of technology was particularly well aligned with our research questions and sample. The framework includes six constructs: individual computer self-efficacy, technology characteristics, task complexity, execution sequence attractiveness, feedback inquiry, and performance. In addition to the more straightforward concepts of computer self-efficacy, task complexity, and feedback inquiry, Goodhue (31) elaborated on the more complex components of technology characteristics, execution sequence attractiveness, and performance. Technology characteristics are composed of three dimensions: technology reliability, usefulness, and complexity. Execution sequence attractiveness refers to the assessment of effort required relative to the quality of output when considering the use of new technology. Simply put, this concept can be summarized by the following question: “Is it worth it?” Finally, Goodhue (31) considered performance to represent improved efficiency and effectiveness as a result of task–technology fit. In addition to questions specifically related to TTF, we incorporated practical questions to better understand what SFD programs are currently doing with virtual coaching education, how they have chosen which technologies to adopt, and their general impressions of the effectiveness of virtual coach training.

When initially contacted about the study, participants received a description of the study, including the ethics board approval, along with an information sheet outlining confidentiality, risks, and benefits. At the start of each interview, conducted in English via Zoom, the researchers obtained verbal consent, ensuring that participants had reviewed the information sheet and were confident proceeding with the interview. Interviews were conducted between January and June 2025, lasting an average of 42 min (ranging from 34:13 to 54:30 min). Interviews were recorded via Zoom’s built-in audio recording and auto-transcription features. Transcriptions were first checked for accuracy by the research team and then sent to the participant interviewed for verification.

### 2.1 Analysis

As the interview questions were both theory (Task–Technology Fit) and non-theory based, data analysis took a hybrid deductive/inductive approach (32). We followed a three-step process of data reduction, data display, and conclusion drawing (33). First, all interviews were transcribed and sent back to the interviewee for a process of member checking. Second, data were deductively analyzed utilizing the constructs from TTF as the primary codes (e.g., technology characteristics) and secondary codes (e.g., technology usefulness). Both members of the research team independently read, analyzed, and coded data from the first six interviews (data reduction). They then worked together to compare coding decisions, discuss emerging patterns, and address any disagreements to ensure alignment in analysis (data display) (28). After establishing consensus on the initial coding, the researchers read each transcript again to confirm the collective themes and undertake inductive analysis to capture any emerging themes not aligned with the TTF codes. The researchers reconvened and removed any TTF codes that were not supported by the data from the analysis. At this stage, data were approaching saturation, as clear themes had begun to emerge. However, the research team decided to add four more SFD organizations to the sample to expand the geographic diversity of the sample and to confirm that saturation had been reached. After adding the final four interviews (bringing the sample size to a total of 10), data analysis was repeated in the same manner for all ten interviews, ending in conclusion drawing. In alignment with our study's post-positivist approach, our analysis process was an iterative one in which inter-coder comparison and data revisiting were undertaken to uphold methodical rigor and reduce the innate subjectivity in the analysis process (33).

### 2.2 Positionality

Data analysis was undertaken with the understanding that researchers construct meaning from the data throughout the process and that they can never truly be neutral observers. This perspective aligns with the study’s post-positivist approach: “Each one of us lives out our lives in the context of a worldview, which influences how we think and behave and how we organise our lives, including how we approach research” (29). In consideration of this, the researchers acknowledged their positionality and its influence on the project. Both members of the research team are SFD academic scholars, who have additionally been involved in directly running or implementing SFD programming for 18 and 8 years, respectively. They are both white women born and raised in the United States, both of whom have lived abroad in the Global South. Both of them worked with SFD organizations as researchers and practitioners in their home country and across the Global South, collectively in 15 countries. They brought this perspective to this study, including their personal experience running both in-person and virtual training for SFD coaches. The research team discussed these experiences before collecting data, understanding both the strengths and challenges of having direct connections to the work. Throughout the interview process, the team was able to deepen their understanding of the context and ask critical follow-up questions relevant to the work. During the data analysis phase, particularly when engaging with collective themes analysis, the team repeatedly returned to the data to ensure that their own experiences did not impact results and but that the themes were derived explicitly from the interviews themselves.

## 3 Results

Four major themes were identified through the research process: two themes aligned with the TTF and two additional themes emerged through inductive analysis. The four themes include attractiveness/motivation (related to Execution Sequence Attractiveness in TTF), utility (related to Technological Characteristics in TTF), access/resources, and pedagogy/method of delivery. Table 2 outlines the identified theme, indicating which were aligned with the TTF model and which were not.

**Table 2:** Major themes and task–technology fit alignment.

Theme/finding	Aligned TTF construct
Utility →	Technology characteristics
Attractiveness/motivation →	Execution sequence attractiveness
Access/resources	
Pedagogy/method of delivery	

No theme alignment with the following TTF constructs: individual computer self-efficacy, task complexity, feedback inquiry, performance.

### 3.1 Attractiveness/motivation

The first theme related to the attractiveness of virtual technology and the motivation of organizations to utilize virtual methods for coach education. Grouped under the heading of Execution Sequence Attractiveness in the framework (31), the category reflects participants’ consideration of the effort required to adopt new technology and its ability to improve effectiveness. Generally, our sample was highly motivated to integrate virtual education, acknowledging its potential value. Tim (North America) shared, “And I've had to, as I was kicking and screaming into the virtual world, I've realized that this is a tool. This is not going away. And therefore, because it's not going away, we have to get better at using it.” John (Central Africa) added, “…virtual training could be something very, very important but also it doesn't come alone like that. There's just a lot of planning and resources that need to be allocated for that…but it's something now we can bring in to be like, hey, we need to do this.”

Despite this motivation and acknowledgement of the realities of our technologically advancing world, many participants struggled with questions of how best to use it and the effort required to make it effective. Their reflections often ended with the sentiment “Is it worth it?” Participants expressed the following:

I think the number one issue is noise. It's always noise, particularly when you are trying to work with a large group of people. It's the muting and the unmuting and the, you know, the time lag with that it just really disrupts the flow. And for me, like I said, I'm a very expressive trainer … I mean, it's crazy. How much of an impact it makes in the flow … because you'll make this really like important point. And you're so energetic about it. And then it's just silence (Lorie, Southeast Asia).

… like virtual learning I think is good, but I just think there needs to be some kind of homework or applied, really simple way for them to practice it. And then there just has to be follow through. But I don't know, you know, honestly, I don't know if that is just more hassle than it's worth (Gail, Sub-Saharan Africa).

Even with these challenges, several participants continually expressed motivation to try virtual education due to the myriad of benefits they perceived for their organization, and the ways that this technology could help them in being more effective in how they supported their coaches as well as increasing access to learning resources. David (Sub-Saharan Africa) stated the following:

I think virtual, I mean for us, and what we consider makes us a strong organization, is even though we're extremely, like, locally-focused, we are plugged into the global conversation. Virtual is critical because, I mean, it expands the world beyond what other organizations in our community are doing.

… I think because of the learning culture that we've created, people get more hungry to learn. And then it's like, well, we have limited resources to, you know, we don't have enough money to access it for you … So yeah, I think there's a lot once you kind of get the right people with the right mindset and they just get hungry to learn … So yeah, I would just say there's an endless amount of things and we've been fortunate to access some of them.

While participants voiced motivation to utilize virtual education, when presented with a choice between in-person and virtual training, they often expressed a preference for in-person training due to what they believed was a better training experience. Gail (Sub-Saharan Africa) shared, “… If someone were to give me an option. Do you want to train virtually for 2 weeks at home, or do you wanna travel 48 h and lose all your sleep and get sick, and, you know, go to a different country and train someone in person. I would still choose training someone in person.”

### 3.2 Utility

The second theme was utility, which fell under the classification of technological characteristics in the TTF framework. In this theme, participants described multiple ways in which virtual technology was integrated into the overall strategies for coach education and organizational capacity building. Participants also described using virtual technology for initial meetings with coaches, as a hook for larger in-person training sessions, as touch points or informal follow-ups after in-person trainings, for sharing static content, and for building a community and supporting a culture of learning.

Adam (Europe) described his organization's use of virtual technology in two key ways: initiating relationships and building momentum. His organization sees the virtual space as a place to engage in broader global conversations and bring together partners who might not otherwise interact outside of this environment. He shares the following:

And there's more and more opportunity for folks that are investing in capacity building to do the virtual first and sort of test it out a little bit. And then from there we hope to sort of build the momentum of what's happening in the virtual space and translate it. But a lot of the content is very much the same. It's just delivered differently based on the sort of the mechanism…

… when it comes to our work it's part of growing the movement … It allows that space to get creative and it also allows us to build up like a database, or like a list of organizations that are interested in this. So as we get into more funding conversations, or, as we think about how we're building coalitions and local communities, that sort of hour on Zoom (or whatever it is) can really keep that ball rolling.

Virtual technology was also useful to facilitate informal check-ins, particularly when organizations had coaches placed across the country and in-person meetings were both logistically and financially not feasible. Several of the organizations interviewed operate across a larger geographic area within a country and cannot always bring all the coaches together for trainings. The virtual environment allows them to supplement the in-person trainings with follow-ups and more regular check-ins. Riyaan (South Central Asia) describes it in the following manner:

…in-person things happen every weekend on Saturday. They come and they refresh what they are going to deliver the next week. But this cannot happen with the staff from farther away. So we do the same over Zoom for them. So someone who's running the program in a more remote location is very well aware of the content that needs to be delivered in the next week. So they do a virtual training session with them online, an hour session. It's not very deep because we've already taught them how a sample session looks like. So what we're trying to get from them over this one hour call is if they have clarity regarding what needs to be delivered and will it be age appropriate with the examples that they're using, the language that they're using, is it at the level with where the participants are.

Tim (North America) shared a similar sentiment, in that the best utilization of virtual coach training is as supplemental training that can happen in between the times when the coaches can get together in person. While the content delivered may be different in the two environments, the virtual sessions allow the coaching education to maintain its momentum in between in-person sessions. Importantly, every SFD organization interviewed noted that virtual coaching education, at this point, is purely supplemental to in-person. Tim stated the following:

Ideally, oh, it'd be great if we had the first interaction in person and then we can go to the online. We have two meetings first in a virtual space. And then this will be in person, the next one. And I guarantee, it never fails, that the next three months after this weekend's meetings with all the coaches, that is going to be way better, higher level of interaction, higher level of participation and quality of work compared to these past two and a half months coming into this in person, because they've had an opportunity to build a rapport with myself and the other instructor.

Although participants described a variety of ways in which technology was useful, they were quite also about circumstances in which they would or would not engage in the virtual space. Their choice was shaped by the sensitivity of the topics being addressed, building new relationships, or the inaccessibility of virtual platforms for some partners. There appeared to be a clear intentionality when choosing to use virtual technology. Jordan (Oceania) shared the following:

The solutions need to be a lot more localized, based on the strengths and the resources that exist locally. And so for us, needing to deeply understand well what is happening locally? What might be the needs of learning, development, training? And then how might we tailor workshops or content that really supports meaningful learning for that particular local group. That's a big part of our approach now. There's more, I guess, authentic engagement that needs to happen.

### 3.3 Access and resources

The third theme was related to access and resources, which were often discussed as challenges to technological use within organization and among coaches. There were several respondents who pointed to a lack of “in-country” resources when it came to coach education, professional development, and training curriculum. This was highlighted as the rationale for using virtual coach education, but it often signaled a larger, underlying challenge related to the socioeconomic environment in which organizations were operating. In these instances, participants described a myriad of barriers that this presented to virtual education. Riyaan (South Central Asia) reported the following:

So one thing we don't have access to is professional coaches, coach trainers. Like we are doing the best we can with whatever we access or the resources we get. I have several friends who continue to guide me online, may come once a year or twice a year, but this is still less. When you want to develop coaches professionally, there needs to be some resource person who is there at a much higher level than what your coaches are. That is definitely one big need that we feel right now.

Two participants shared this same sentiment about their respective global regions. David (Sub-Saharan Africa) explained: “… the learning opportunities are really, like, few and far between. I mean, in all the time that we've been working here, which is almost 12 years, they've hosted a course in this region, like probably less than four or five times. Whereas like in the US Soccer Federation, it's happening all the time everywhere.” Gail (Sub-Saharan Africa) added the following:

I just think that the coach education, the coach training in these countries is just so low that there isn't, like, you can't pull from within. So yeah, you pull externally. And that's what I think we've tried to do through partnerships and whatnot is to bring that in from outside the country. And if it is somewhat virtual, I still think that that's understanding how to be a really good coach, even sitting in a session is important.

Nevertheless, the opportunity to address gaps in professional coach training was significantly marred by the overwhelming challenges associated with virtual education as a result of poor internet connectivity, data usage charges, transporting coaches to a central location for a session, and financial fees to gain access to some virtual materials. Lorie (Southeast Asia) highlighted the logistical challenges faced by virtual coaches working in remote communities: “And there's just so many logistical issues with virtual anything when you're working really rurally, that it seems to be more of a distraction than anything. If the internet's constantly going out, and you can't hear. I think with more experienced people it can be used effectively. But I think it takes a while and a lot of time to get to that point.”

Tim (North America) elaborated on these challenges of accessibility as a result of a lack of internet access and high cost:

Accessibility, you know, it's interesting that that question is such a big one from the standpoint of, you know, where we live. In a lot of this area where the children do not have internet in their homes, right? So I guess accessibility, even geographic, we'll have to look at that…some of the virtual options are not accessible either because they don't have internet or they even do cost money. And that's a barrier for a lot. Even a hundred dollar course is quite a barrier for a lot of people who want to get better at coaching.

Lastly, participants recounted the struggles they faced when setting up online sessions for their coaches, with some (e.g., Riyaan) ultimately shifting back to in-person formats to overcome these challenges. Lorie (Southeast Asia) explained, “… It was really, really challenging. Because, you know, we worked in very rural areas and found that most houses had a smartphone right? And then, second, of course, there's never network. And then there's never air time.” Riyaan (South Central Asia) also noted the following:

… It's also having enough data to attend these types of calls. So each coach is like attending from their own phone and they don’t have enough data. So we've tried to address this by asking them to come to our office. They don't have to waste their data. And since all of the coaches live in the very same community, like 10 or 15 min away from the office, it's easier for them to get there on a Saturday morning …. But these are all the real concerns like data size, data consumption, bandwidth issues, software issues. These are the three things which you will struggle with when you initially start with online sessions.

### 3.4 Pedagogy/method of delivery

The fourth and final theme was pedagogy/method of delivery, which encompassed the varying ways the participants and their respective organizations approached teaching in a virtual space and how best to support learning. Strategies included explicit facilitation, adapting delivery, and modeling behavior in a virtual environment. Across responses, a common thread was a focus on building connections and engagement.

One important concept that emerged was the need to think intentionally about engagement in the virtual space, rather than simply replicating in-person approaches. Several participants emphasized that online coach education required distinct strategies to make it effective, highlighting both challenges and the effort required. Jordan (Oceania) noted the need for well-trained online facilitators:

Virtually, you know that some can really entrench themselves in in a virtual world, whereas others being in front of the screen for prolonged periods of time, isn't their jam as well, and the balance between information push, (i.e., them sitting there, being talked at and spoken at vs. being in a virtual room, being in breakout rooms, and being able to converse with others). We found that there's a real positive balance, and the more facilitated approach, and supporting them to be pushed into breakout rooms being facilitated by a person where everyone's sharing space, everyone's sharing the microphone and speaking about whatever the concept or the topic is… it does feel like a need for facilitation, more, more explicit facilitation.

Tim (North America) elaborated on how he had adapted his previous teaching style to better fit the virtual space in order to promote greater buy-in among coaches:

What I've had to learn, this is what I'm trying to get better at, is come up with learning activities within this virtual space so that it's not an information push. Very easy to come up with PowerPoints and just click…But when we start designing things, whether it's through Google Sheets and Google Forms, where we can have breakout rooms and going into an area and then you and I as leaders popping into group one, two, three, and then popping into group x,y,z to talk about things. And we could really reach more people that way and actually quite frankly have buy-in, right?

Further considering the personal strategies of trainers in the virtual space, Adam (Europe) reflected on the following:

I think the crux of all of it for us is, what's the experience? How are we modeling? So I think, for us, the transferable of modeling. The practice is obviously much harder virtually, and so I think we put more emphasis on that. And so how are we building relationships in a virtual room? What are we intentionally doing? How are the activities? Are they a safe space to take risk? How are they engaging in a way where it's meeting different styles of learners and different styles of risk-taker and challenge?

Similarly, participants discussed the ongoing challenges of engagement in a virtual environment, which, at times, can be a more sanitized space for learning and relationship-building. Within the field of coach education in particular, many emphasized that coaches typically preferred face-to-face interactions as they play such an integral role in their profession. Riyaan (South Central Asia) recalls, “Especially when it comes to coaching, this is definitely more in person because it's very physical. So you can teach the theory, but definitely there needs to be some clarity with regards to what happens on the field.” Others, such as Adam (Europe), shared similar challenges:

I think Covid played a little bit of a role in normalizing virtual training. More often we pretty much always say, like, you can't replace the in-person energy. And you know that we're very much activities based. And kind of doing some concepts, doing some sort of theory and then practicing that theory and then applying it, especially if we're in like a sports specific place.

Lorie (Southeast Asia) added, “I think virtually it feels like a lot is missing in well, obviously, body language, it's far harder to tell virtually, if someone is lost, I think they hide it much better when you do it virtually. It's also really harder to draw out engagement virtually in my experience. It's kind of ‘anyone have any questions?’ and no one says a word.”

Finally, several respondents outlined a hybrid approach, in which the virtual education was used as a supplement to in-person training. David (Sub-Saharan Africa) explained, “So the hybrid model works well for us where we watch the content together and then we discuss it in-person and then we can contextualize it as well. So that's what we have right now is our onboarding process internally, coaching, soccer coaching, education, external, and then with those virtual partnerships to supplement the weekly learning.”

As mentioned previously, David (Sub-Saharan Africa) and others saw virtual coaching education as a supplement to in-person training. They felt that the virtual space worked much better when in-person connections had already been made, because the trust needed in coaching, mentoring, and education had already been established. Lorie (Southeast Asia) noted the following:

I would feel far more comfortable doing it with coaches I've met, because I know them, and I've met them in person than those I've never met in person. I think just, you know, being able to call out a name, and you know, put someone on the spot that I see not paying attention or something. I know that's like the negative side of it, but just being able to actually engage with a person that I've seen before and met, I feel like that would create more of a connection through a computer screen which is hard to create a connection through.

## 4 Discussion

While this study was not undertaken as a comparison of the Global North and Global South, some important distinctions did arise. Four of the organizations interviewed are based in Western or Global North countries, while the other six are located within the Global South. The Global South broadly refers to Latin America, Asia, Africa, and Oceania and is often associated with generalizations such as marginalized and low-income communities (34). The terms are often oversimplified and unrepresentative (35), and as Patrick and Huggins note, the Global South has “become a convenient shorthand for a broad swath of nations seeking to overhaul the unjust structures of the global economy” (36). Further, this classification does not represent the heterogeneity of the region. Nevertheless, within the current study, noteworthy differences based on economy and privilege did emerge.

While organizations across all locations celebrated their highly motivated coaches who are eager to access coaching education in any form, those located in rural and remote communities faced significant barriers to doing so. Organizations noted challenges such as the high cost of cellular or Wi-Fi data, the unpredictability of the internet (even in locations where they seemingly have access), the logistics required to bring coaches to a central location for trainings or Zoom/Teams calls, and language barriers. While the knowledge, attitudes, and motivation of coaches were high—factors that have been shown to contribute to successful outcomes in those they coach (10, 16)—these strengths were not sufficient to overcome the real logistical challenges of accessing virtual coach training. Several scholars have noted similar findings in terms of geographic locations, inaccessibility, lack of local expertise, and financial constraints being limiting factors in the impact of coaching education (19–22).

Meanwhile, in North America and Europe, accessing virtual training opportunities was relatively cheap and simple and placed very few resource constraints on the SFD organizations. Coaches in SFD organizations across the Global North were able to tap into a variety of virtual training types—videos posted online, live sessions, and even tailor-made curriculum delivered remotely. Several conclusions can be drawn from this, the most pressing of which relates to equity.

It is true that we have never been a more connected world, thanks in large part to technological advancements. The benefits to virtual or online learning include its relatively low cost, wide reach, and accessibility (24). However, the mere existence of technology does not make it accessible and usable for all. Organizations doing similar work, in very similar ways, experience different barriers to access that exacerbate the equity gap. In this study, SFD coaches in Europe and North America were able to access a myriad of virtual training opportunities, with the only real barrier being a cost in the case of paid programs/subscriptions. In Africa, Asia, and Oceania, cost was but one of the barriers faced. Even if the organizations were able to overcome that, there were many more obstacles to overcome for their coaches to engage in virtual spaces, including poor internet connectivity, limited data availability, and at times transportation of coaches to a central location for a virtual session. As many SFD organizations are grassroots organizations that are under-staffed, under-resourced, and located in the Global South (37–39), the ease or difficulty with which coaches can access virtual learning impacts the result. Is the outcome worth the effort?

In short, the current study revealed a major equity issue in relation to SFD organizations engaging in virtual coaching training. Those who already had access to relatively sophisticated training programs, master coaches, and resources were also the ones who were most frequently engaged in the virtual learning spaces. On the contrary, those who had the greatest need for virtual training still faced too many barriers to utilizing it to its full potential. So, while many believe that virtual coaching education is a strong solution in rural communities where there might not be as many in-person opportunities offered, this study found that not to be the case. Rather, virtual inaccessibility could be contributing to a widening in the equity gap. This is especially concerning given the literature that affirms how important coaching education is in successful programming (9, 16).

Though a lack of equity in access was apparent, the findings were not entirely discouraging. As Hans Rosling stated in *Factfulness* (35), the reality is both bad and better. While barriers to access, especially in rural or remote communities, were still highly problematic, respondents were also quick to note that the situation was improving. Many expressed optimism that this trend will continue, enabling coaches in remote communities to easily access virtual coach training with the same ease as those in other countries already can. But why wait? Why not think more creatively now, rather than just wait for things to change? Across the SFD organizations interviewed in this study, virtual coach training was most often conceptualized as live sessions (Zoom/Teams) or video tutorials. These strategies are easily accessible, given the widespread use of virtual education in the Global North, especially given familiarity with these methods of delivery following the pandemic (23). Coaching organizations continue to utilize online learning (24, 25), but likely in the same standard ways. Although these “traditional” methods are ineffective in rural and remote communities, we have not moved beyond them. It would be a worthwhile endeavor to bring together SFD practitioners to brainstorm or engage in design-thinking around what access to the global conversations on coach training might look like in more non-traditional ways. How can we tap into improved technology and virtual coach training in ways that have not yet been suggested? What other disciplines might we look to for guidance that have found success in more accessible forms of remote learning that we can utilize now? Insights from broader digital inclusion literature highlight practical enablers to address existing issues of equity, including device sharing, data stipends, and community hubs (40). In addition, low-bandwidth models (such as asynchronous micro-learning via WhatsApp or SMS, downloadable packs, radio options, or local television broadcasts), community-based peer facilitation, and other hybrid pathways are ideas worth exploring.

As an example of thinking beyond Zoom or Teams, two participants interviewed reported attempts their organization had made to offer virtual programming that is more accessible or creative for those in rural areas. One shared, “One of my deliverables that I got to do is create an online free course so that people can log in and take this course for free. We do this course for free in a bunch of those different communities that just don't have the financial resources.” Another participant discussed a “virtual classroom” that was created via a WhatsApp group. In this model, the program recorded short (2 min) videos for the coaches to watch, followed by opportunities to engage and ask questions within the WhatsApp group chat. While initial engagement was high (particularly when the internet connection was reliable), it dropped after a few weeks, with fewer coaches participating. In the participant’s own words, “It would start really well. So the 1st day, the second day, the 3rd day, we'd have 45–50 signing in by the 3rd day we're down to like 30, and then it would continue to trickle off. And it was just that, maintaining the interest in it was really difficult.”

Although neither of these strategies seemed to completely hit the mark, they do demonstrate that SFD organizations are attempting to find more creative or usable strategies for virtual coaching education beyond videos and Zoom/Teams live sessions. SFD organizations are forced to think creatively or “outside the box” regularly, given their often low-resourced functioning (41, 42). However, it appears that this “out of the box” thinking has yet to be applied to virtual coach education. More creative thinking could lead to better ways of utilizing online learning now, while also anticipating future advances in technology and data connectivity in rural settings. It would also be prudent to utilize more creative thinking around coaching education broadly. Are there other barriers (besides technology) that are preventing coaches from fully engaging in the opportunities offered? Although administrators reported that coaches have a high level of motivation for training, some of the behaviors do not necessarily demonstrate this (such as the WhatsApp group described earlier that had high engagement initially, then a sharp decline). Extending this line of enquiry to include conversations directly with coaches would provide valuable insights into their perspectives. Such dialogue could help identify alternative approaches and foster more creative solutions between administrators and coaches.

Given the utilization of Task–Technology Fit as a framework for this study, it is also worth discussing its value here. While there was certainly a value added in the development stage of the study, in framing questions around the different aspects of TTF, our overall assessment is that most of the organizations interviewed are still in their early stages of tapping into technology for coaching education and perhaps have yet to reach a minimum threshold of use to be able to apply TTF. For instance, because of the many barriers faced related to connectivity, resource allocation, and access, most of the organizations were just starting to tap into virtual coach training, and in very basic ways (recorded videos, Zoom or Teams calls, etc.). In many cases, the organizations were accessing the low-hanging fruit of virtual technology for coach training, as most of them were not assessing fit in an intentional way. Task–Technology Fit proposes that users assess aspects including computer self-efficacy, task complexity, feedback loops, effort, quality, and performance (43). These are in addition to the technology characteristics of reliability, usefulness, and complexity. Most organizations interviewed for this study had not yet moved beyond the technology characteristics in their use of virtual coach training. Therefore, for this theory to be most usefully applied, organizations need to meet a minimum usage threshold—one that we did not consider our participants to have met. This raises important issues for future enquiry: When and how do organizations reach a threshold of use for the theory to be able to apply more fully?

In conclusion, it is important to note that none of the organizations interviewed as part of this study explicitly or systematically assessed the effectiveness of their virtual training programs, and information regarding their programs was self-reported without behavioral or learning outcomes. Therefore, any comments on what is or is not working are anecdotal. Li et al. (16) conducted a meta-analysis of the effectiveness of coaching education, but did not distinguish between in person or online, finding that most programs result in better coaching. Meanwhile, Svensson and Woods stated that “Even well-designed SDP initiatives may not necessarily result in positive outcomes” (39). Both these studies indicate that further research and analysis are required in the field of coaching education in SDP. There is a need to more intentionally assess SFD-focused coaching education programs in a way that measures both in-person and online/virtual strategies, so that we can better understand the value each provides.

Future research could benefit from various topic and methodological directions to deepen our understanding in this field. Promising topics include the closer examination of program delivery, content, and curriculum—with particular attention to emerging critiques of traditional coach education—and the transfer of knowledge under a social justice lens (44). There was also an assumption made in this study that coaches want more coaching education and possess the motivation for it. However, further reflection on the purpose of coaching education and what topics are included could be highly valuable. For instance, is knowledge what coaches most need in coaching education, or are there additional—or more important—elements that should be included, such as creating safe spaces, fostering inclusion, and sustaining motivation? Methodologically, subsequent studies could strengthen rigor through data triangulation—incorporating observational methods, pre- and post-assessments, and the inclusion of coaches as key participants in the research process. Our goal is to continue to better understand this subject, contribute to it both practically and theoretically, and help improve the long-term impact of SFD programming.

## Data Availability

The raw data supporting the conclusions of this article will be made available by the authors, without undue reservation.
